# (Butane-1,2,3,4-tetraol-κ^3^ *O* ^1^,*O* ^2^,*O* ^3^)(ethanol-κ*O*)tris­(nitrato-κ^2^ *O*,*O*′)erbium(III)

**DOI:** 10.1107/S1600536813008003

**Published:** 2013-04-13

**Authors:** Xiao-Hui Hua, Jun-Hui Xue, Li-Min Yang, Yi-Zhuang Xu, Jin-Guang Wu

**Affiliations:** a Beijing National Laboratory for Molecular Sciences, The State Key Laboratory of Rare Earth Materials Chemistry and Applications, College of Chemistry and Molecular Engineering, Peking University, Beijing, People’s Republic of China; b Chemical Engineering College, Inner Mongolia University of Technology, People’s Republic of China; c State Key Laboratory of Nuclear Physics and Technology, Institute of Heavy Ion Physics, School of Physics, Peking University, Beijing, People’s Republic of China

## Abstract

In the title Er^III^–erythritol complex, [Er(NO_3_)_3_(C_2_H_5_OH)(C_4_H_10_O_4_)], the Er^III^ cation is chelated by one erythritol mol­ecule, three nitrate anions and an ethanol mol­ecule, completing an irregular ErO_10_ coordination geometry. The Er—O bond lengths are in the range 2.348 (3)–2.583 (3) Å. In the crystal, extensive O—H⋯O hydrogen bonding links the mol­ecules into a three-dimensional supra­molecular structure.

## Related literature

For crystal structures of related lanthanide nitrate–erythritol complexes, see: Gyurcsik & Nagy (2000 ▶); Yang *et al.* (2003 ▶, 2004 ▶, 2012 ▶). For the isotypic Ho^III^ complex, see: Hua *et al.* (2013 ▶). For the structure of erythritol, see: Bekoe & Powell (1959 ▶).
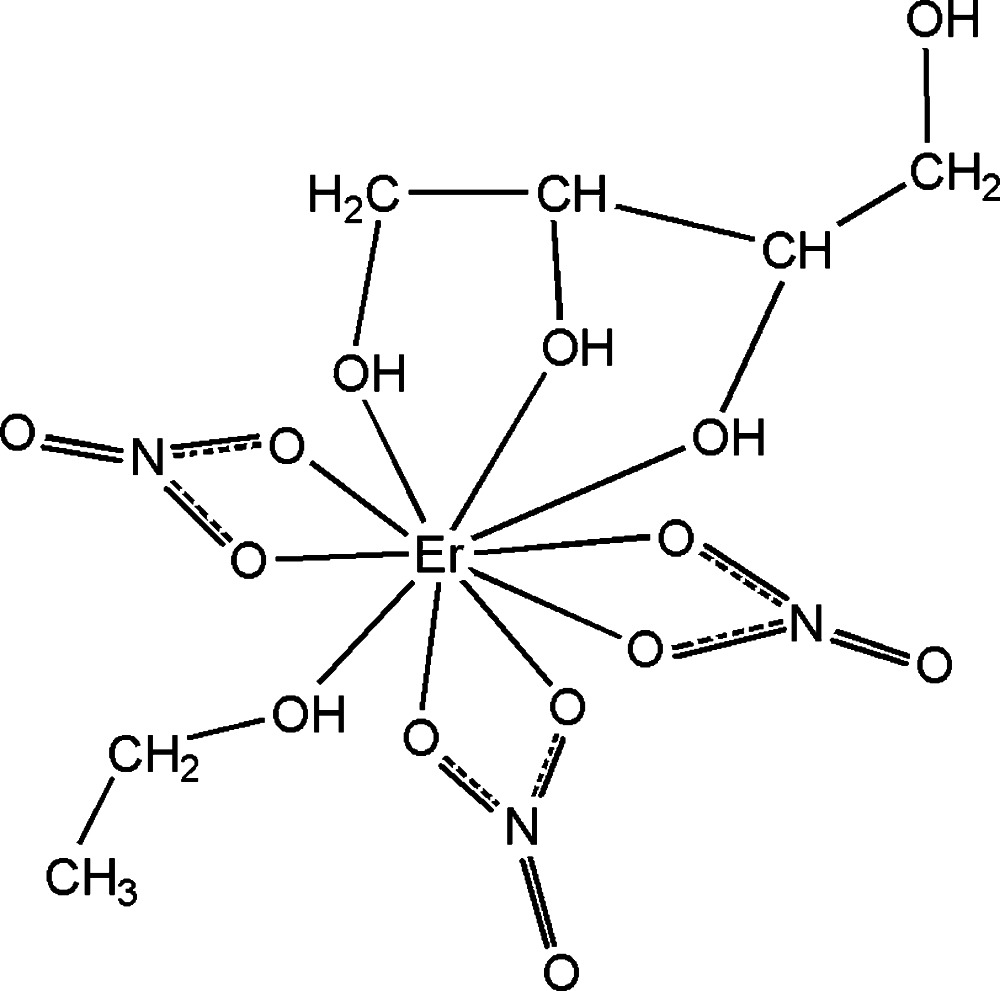


## Experimental

### 

#### Crystal data

[Er(NO_3_)_3_(C_2_H_6_O)(C_4_H_10_O_4_)]
*M* *_r_* = 521.48
Monoclinic, 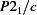

*a* = 7.7521 (16) Å
*b* = 12.772 (3) Å
*c* = 15.121 (3) Å
β = 100.26 (3)°
*V* = 1473.3 (5) Å^3^
*Z* = 4
Mo *K*α radiation
μ = 5.78 mm^−1^
*T* = 173 K
0.23 × 0.20 × 0.06 mm

#### Data collection

Rigaku Saturn724+ CCD diffractometer
Absorption correction: multi-scan (*CrystalClear*; Rigaku, 2007 ▶) *T*_min_ = 0.12, *T*_max_ = 0.35
10846 measured reflections
3359 independent reflections
3174 reflections with *I* > 2σ(*I*)
*R*_int_ = 0.035

#### Refinement

*R*[*F* ^2^ > 2σ(*F* ^2^)] = 0.031
*wR*(*F* ^2^) = 0.063
*S* = 1.20
3359 reflections
218 parameters
H-atom parameters constrained
Δρ_max_ = 1.46 e Å^−3^
Δρ_min_ = −0.62 e Å^−3^

### 

Data collection: *CrystalClear* (Rigaku, 2007 ▶); cell refinement: *CrystalClear*; data reduction: *CrystalClear*; program(s) used to solve structure: *SHELXTL* (Sheldrick, 2008 ▶); program(s) used to refine structure: *SHELXTL*; molecular graphics: *SHELXTL*; software used to prepare material for publication: *SHELXTL*.

## Supplementary Material

Crystal structure: contains datablock(s) global, I. DOI: 10.1107/S1600536813008003/xu5657sup1.cif

Structure factors: contains datablock(s) I. DOI: 10.1107/S1600536813008003/xu5657Isup2.hkl

Supplementary material file. DOI: 10.1107/S1600536813008003/xu5657Isup3.cdx

Additional supplementary materials:  crystallographic information; 3D view; checkCIF report

## Figures and Tables

**Table 1:** Selected bond lengths (Å)

Er1—O1	2.348 (3)
Er1—O2	2.368 (3)
Er1—O3	2.463 (3)
Er1—O5	2.352 (3)
Er1—O6	2.438 (3)
Er1—O7	2.434 (3)
Er1—O9	2.583 (3)
Er1—O10	2.428 (3)
Er1—O12	2.436 (3)
Er1—O13	2.489 (3)

**Table 2:** Hydrogen-bond geometry (Å, °)

*D*—H⋯*A*	*D*—H	H⋯*A*	*D*⋯*A*	*D*—H⋯*A*
O1—H1⋯O4^i^	0.84	1.83	2.666 (4)	173
O2—H2⋯O9^ii^	0.84	1.97	2.795 (4)	167
O3—H3⋯O13^iii^	0.84	2.08	2.917 (4)	175
O4—H4⋯O11^iv^	0.84	2.07	2.897 (5)	169
O5—H5⋯O8^v^	0.84	2.09	2.865 (4)	154

Symmetry codes: (i) 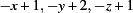; (ii) 
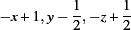
; (iii) 
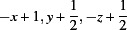
; (iv) 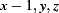; (v) 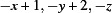.

**Fig. 1.**
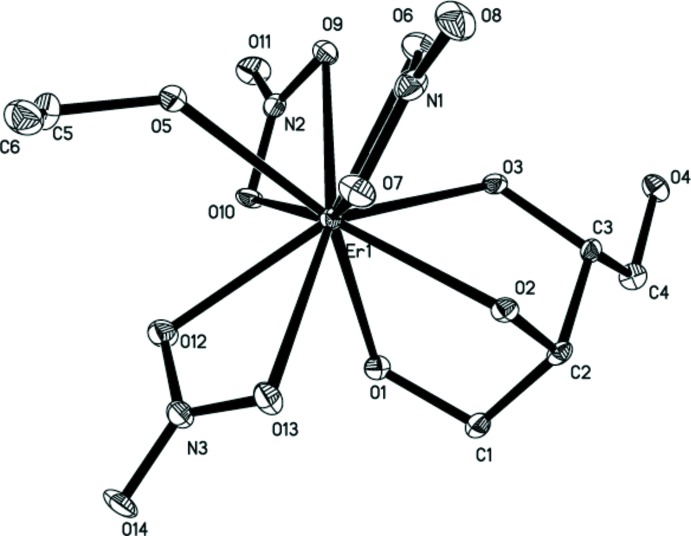
The crystal structure of the title complex, displacement ellipsoids drawn at 30% probability level. The Hydrogen atoms have been omitted for clarity.

## supplementary crystallographic information

### Comment

Sugar-metal interaction is involved in many important biological processes (Gyurcsik & Nagy, 2000). Erythritol was used as a model compound to study the coordination behavior of hydroxyl groups of carbohydrate to metal ions.

The crystal structure of the title complex denoted as ErEN, where E stands for erythritol and N stands for nitrate) is shown in Fig. 1. This is isostructural with the Ho^III^ compex (Hua *et al.*, 2013). Three hydroxyl groups from one erythritol molecule, one hydroxyl group from ethanol, and six oxygen atoms from three bidentate nitrate ions are coordinated to Er(III), making the coordination number 10. Erythritol molecule is an O1, O2, O3-three hydroxyl group donor here.

The structure of ErEN is similar to NdEN, EuEN, YEN, GdEN and TbEN (Yang *et al.*, 2003, 2004, 2012). Er-O distances range from 2.348 to 2.583' Å, the average Er-O distance is 2.419Å. The structure of erythritol changed somewhat in the complex. The C-C bond length is 1.51Å and the C-O bond lengths are 1.39 and 1.47Å for a free erythritol (Bekoe & Powell, 1959). After coordination, the C-C bond lengths are 1.505 and 1.512Å and the C-O bond lengths are 1.422, 1.451, 1.445 and 1.456Å in ErEN. The C-C-C bond angle is 113° and the O-C-C bond angle is 107° for erythritol (Bekoe & Powell, 1959). After coordination, the C-C-C bond angles are 116.3 and 113.0° and the O-C-C bond angles range from 103.6 to 111.7° in ErEN. In addition, the torsion angle of C-C-C-C is 180° for erythritol. After coordination, the torsion angle of C-C-C-C is -57.2 (4)° in ErEN. The variation of the C-C-C-C torsion angle indicates the coordination to Er^3^^+^ brings about significant variation of the conformation of erythritol.

The hydrogen bond networks in ErEN are formed by O—H···O hydrogen bonds between coordinated and uncoordinated hydroxyl groups of erythritol, ethanol and nitrate ions.

### Experimental

Er(NO_3_)_3_.6H_2_O and Erythritol were purchased from Shanghai Aladdin Chemical Reagents Company and was used without further purification. The procedure for the preparation of the title compound is as follows: Er(NO_3_)_3_.6H_2_O (3 mmol) and erythritol (3 mmol) were dissolved in 6ml water and 6 ml ethanol. The solution was put on a water bath, and the temperature was raised to 353 K. Small aliquots of EtOH were periodically added to the solution during the heating process to prolong the reaction time. The resulting mixtures were filtered and left for crystallization in room temperature, the suitable crystals for X-ray diffraction measurements were obtained in two weeks.

### Refinement

The C-bound H-atoms were placed in calculated positions (C—H 0.930 Å) and were included in the refinement in the riding model approximation, U_iso_(H) = 1.2U_eq_(C). The O-bound H atoms were located in a difference Fourier map and were refined with distance restraint of O—H = 0.84 Å, U_iso_(H) = 1.2U_eq_(O).

### Crystal data

**Table:**

[Er(NO_3_)_3_(C_2_H_6_O)(C_4_H_10_O_4_)]	*F*(000) = 1012
*M**_r_* = 521.48	*D*_x_ = 2.351 Mg m^−^^3^
Monoclinic, *P*2_1_/*c*	Mo *K*α radiation, λ = 0.71073 Å
Hall symbol: -P 2ybc	Cell parameters from 5453 reflections
*a* = 7.7521 (16) Å	θ = 2.1–27.5°
*b* = 12.772 (3) Å	µ = 5.78 mm^−^^1^
*c* = 15.121 (3) Å	*T* = 173 K
β = 100.26 (3)°	Plate, pink
*V* = 1473.3 (5) Å^3^	0.23 × 0.20 × 0.06 mm
*Z* = 4	

### Data collection

**Table:**

Rigaku Saturn724+ CCD diffractometer	3359 independent reflections
Radiation source: fine-focus sealed tube	3174 reflections with *I* > 2σ(*I*)
Graphite monochromator	*R*_int_ = 0.035
Detector resolution: 28.5714 pixels mm^-1^	θ_max_ = 27.5°, θ_min_ = 2.1°
ω scans at fixed χ = 45°	*h* = −10→9
Absorption correction: multi-scan (*CrystalClear*; Rigaku, 2007)	*k* = −16→16
*T*_min_ = 0.12, *T*_max_ = 0.35	*l* = −19→19
10846 measured reflections	

### Refinement

**Table:**

Refinement on *F*^2^	Primary atom site location: structure-invariant direct methods
Least-squares matrix: full	Secondary atom site location: difference Fourier map
*R*[*F*^2^ > 2σ(*F*^2^)] = 0.031	Hydrogen site location: inferred from neighbouring sites
*wR*(*F*^2^) = 0.063	H-atom parameters constrained
*S* = 1.20	*w* = 1/[σ^2^(*F*_o_^2^) + (0.021*P*)^2^ + 2.6512*P*] where *P* = (*F*_o_^2^ + 2*F*_c_^2^)/3
3359 reflections	(Δ/σ)_max_ = 0.002
218 parameters	Δρ_max_ = 1.46 e Å^−^^3^
0 restraints	Δρ_min_ = −0.62 e Å^−^^3^

### Special details

**Table:**

Geometry. All esds (except the esd in the dihedral angle between two l.s. planes) are estimated using the full covariance matrix. The cell esds are taken into account individually in the estimation of esds in distances, angles and torsion angles; correlations between esds in cell parameters are only used when they are defined by crystal symmetry. An approximate (isotropic) treatment of cell esds is used for estimating esds involving l.s. planes.
Refinement. Refinement of F^2^ against ALL reflections. The weighted R-factor wR and goodness of fit S are based on F^2^, conventional R-factors R are based on F, with F set to zero for negative F^2^. The threshold expression of F^2^ > 2sigma(F^2^) is used only for calculating R-factors(gt) etc. and is not relevant to the choice of reflections for refinement. R-factors based on F^2^ are statistically about twice as large as those based on F, and R- factors based on ALL data will be even larger.

### Fractional atomic coordinates and isotropic or equivalent isotropic displacement parameters (Å^2^)

**Table:**

	*x*	*y*	*z*	*U*_iso_*/*U*_eq_	
Er1	0.62537 (2)	0.895819 (13)	0.247063 (10)	0.01275 (7)	
O9	0.6818 (4)	1.0948 (2)	0.26286 (19)	0.0189 (6)	
O13	0.6726 (4)	0.7044 (2)	0.23165 (19)	0.0204 (6)	
O6	0.4323 (4)	0.9877 (2)	0.12708 (19)	0.0228 (6)	
O14	0.9187 (4)	0.6272 (2)	0.2866 (2)	0.0271 (7)	
O10	0.8493 (4)	0.9889 (2)	0.35026 (19)	0.0182 (6)	
O11	0.8904 (4)	1.1563 (2)	0.3659 (2)	0.0265 (7)	
N1	0.4019 (5)	0.9082 (3)	0.0754 (2)	0.0200 (8)	
O7	0.4806 (4)	0.8252 (2)	0.10336 (18)	0.0220 (6)	
O8	0.3011 (4)	0.9117 (3)	0.0031 (2)	0.0280 (7)	
N3	0.8336 (5)	0.7066 (3)	0.2691 (2)	0.0189 (7)	
N2	0.8098 (5)	1.0823 (3)	0.3274 (2)	0.0179 (7)	
O5	0.8049 (4)	0.9494 (2)	0.14542 (19)	0.0206 (6)	
H5	0.7677	1.0027	0.1153	0.025*	
C5	0.9524 (6)	0.9070 (4)	0.1084 (3)	0.0240 (10)	
H5A	1.0284	0.8651	0.1549	0.029*	
H5B	1.0233	0.9653	0.0906	0.029*	
O12	0.8972 (4)	0.7972 (2)	0.2864 (2)	0.0220 (6)	
C6	0.8876 (6)	0.8393 (4)	0.0278 (3)	0.0271 (10)	
H6A	0.8125	0.7837	0.0447	0.041*	
H6B	0.9878	0.8079	0.0063	0.041*	
H6C	0.8201	0.8821	−0.0200	0.041*	
O2	0.3580 (3)	0.8114 (2)	0.25801 (16)	0.0144 (6)	
H2	0.3579	0.7470	0.2467	0.017*	
O1	0.6345 (4)	0.8266 (2)	0.39163 (17)	0.0157 (6)	
H1	0.6813	0.8600	0.4375	0.019*	
O3	0.4312 (4)	0.9935 (2)	0.32999 (17)	0.0156 (6)	
H3	0.3967	1.0527	0.3100	0.019*	
C3	0.2846 (5)	0.9375 (3)	0.3562 (3)	0.0141 (8)	
H3A	0.1758	0.9552	0.3126	0.017*	
C2	0.3211 (5)	0.8219 (3)	0.3484 (2)	0.0155 (8)	
H2A	0.2139	0.7806	0.3540	0.019*	
O4	0.2278 (4)	1.0783 (2)	0.45507 (19)	0.0204 (6)	
H4	0.1250	1.0921	0.4295	0.025*	
C1	0.4774 (5)	0.7790 (3)	0.4124 (3)	0.0188 (8)	
H1A	0.4663	0.7952	0.4752	0.023*	
H1B	0.4832	0.7020	0.4060	0.023*	
C4	0.2584 (6)	0.9690 (3)	0.4493 (3)	0.0191 (8)	
H4A	0.1573	0.9301	0.4648	0.023*	
H4B	0.3637	0.9496	0.4935	0.023*	

### Atomic displacement parameters (Å^2^)

**Table:**

	*U*^11^	*U*^22^	*U*^33^	*U*^12^	*U*^13^	*U*^23^
Er1	0.01351 (10)	0.01084 (10)	0.01385 (10)	0.00053 (7)	0.00234 (7)	0.00034 (6)
O9	0.0162 (14)	0.0161 (15)	0.0238 (14)	0.0023 (12)	0.0024 (12)	0.0028 (11)
O13	0.0159 (14)	0.0188 (16)	0.0254 (15)	0.0029 (13)	0.0006 (12)	−0.0029 (12)
O6	0.0266 (17)	0.0178 (15)	0.0232 (15)	0.0030 (13)	0.0026 (13)	−0.0017 (12)
O14	0.0247 (17)	0.0164 (15)	0.0392 (18)	0.0115 (14)	0.0030 (14)	0.0050 (13)
O10	0.0182 (15)	0.0093 (14)	0.0254 (14)	0.0008 (12)	−0.0006 (12)	0.0031 (11)
O11	0.0318 (18)	0.0123 (15)	0.0330 (17)	−0.0075 (14)	−0.0008 (14)	−0.0056 (13)
N1	0.0213 (19)	0.023 (2)	0.0160 (16)	−0.0027 (16)	0.0049 (14)	0.0022 (14)
O7	0.0269 (16)	0.0203 (16)	0.0181 (14)	0.0069 (14)	0.0023 (12)	0.0006 (12)
O8	0.0251 (17)	0.0375 (19)	0.0188 (15)	0.0062 (15)	−0.0032 (13)	0.0043 (13)
N3	0.0188 (18)	0.0185 (19)	0.0198 (16)	0.0051 (15)	0.0047 (14)	0.0024 (14)
N2	0.0170 (18)	0.0167 (18)	0.0215 (17)	−0.0005 (15)	0.0077 (14)	0.0012 (14)
O5	0.0219 (15)	0.0179 (15)	0.0237 (15)	0.0044 (13)	0.0090 (12)	0.0044 (12)
C5	0.021 (2)	0.028 (2)	0.025 (2)	−0.0008 (19)	0.0086 (18)	−0.0017 (18)
O12	0.0195 (15)	0.0134 (15)	0.0325 (16)	−0.0018 (13)	0.0028 (13)	−0.0011 (12)
C6	0.033 (3)	0.020 (2)	0.028 (2)	0.005 (2)	0.007 (2)	−0.0003 (18)
O2	0.0174 (14)	0.0099 (13)	0.0161 (13)	−0.0012 (11)	0.0032 (11)	−0.0025 (10)
O1	0.0159 (14)	0.0152 (14)	0.0149 (13)	−0.0022 (12)	−0.0001 (11)	−0.0007 (11)
O3	0.0186 (15)	0.0097 (13)	0.0194 (13)	0.0007 (11)	0.0060 (11)	0.0017 (10)
C3	0.0117 (18)	0.0124 (19)	0.0182 (18)	−0.0020 (16)	0.0032 (15)	−0.0011 (15)
C2	0.017 (2)	0.0118 (19)	0.0185 (18)	−0.0049 (16)	0.0055 (15)	−0.0013 (15)
O4	0.0221 (16)	0.0169 (15)	0.0216 (14)	0.0046 (13)	0.0024 (12)	−0.0051 (11)
C1	0.016 (2)	0.018 (2)	0.023 (2)	−0.0033 (17)	0.0034 (16)	0.0031 (16)
C4	0.023 (2)	0.019 (2)	0.0160 (18)	−0.0012 (18)	0.0037 (16)	−0.0030 (16)

### Geometric parameters (Å, º)

**Table:**

Er1—O1	2.348 (3)	C5—H5A	0.9900
Er1—O2	2.368 (3)	C5—H5B	0.9900
Er1—O3	2.463 (3)	C6—H6A	0.9800
Er1—O5	2.352 (3)	C6—H6B	0.9800
Er1—O6	2.438 (3)	C6—H6C	0.9800
Er1—O7	2.434 (3)	O2—C2	1.451 (4)
Er1—O9	2.583 (3)	O2—H2	0.8400
Er1—O10	2.428 (3)	O1—C1	1.445 (5)
Er1—O12	2.436 (3)	O1—H1	0.8400
Er1—O13	2.489 (3)	O3—C3	1.456 (4)
O9—N2	1.271 (5)	O3—H3	0.8400
O13—N3	1.275 (4)	C3—C2	1.512 (5)
O6—N1	1.278 (4)	C3—C4	1.512 (5)
O14—N3	1.212 (4)	C3—H3A	1.0000
O10—N2	1.265 (4)	C2—C1	1.512 (6)
O11—N2	1.223 (5)	C2—H2A	1.0000
N1—O8	1.226 (5)	O4—C4	1.422 (5)
N1—O7	1.259 (5)	O4—H4	0.8400
N3—O12	1.267 (4)	C1—H1A	0.9900
O5—C5	1.464 (5)	C1—H1B	0.9900
O5—H5	0.8401	C4—H4A	0.9900
C5—C6	1.505 (6)	C4—H4B	0.9900
			
O1—Er1—O5	142.67 (10)	O12—N3—O13	115.2 (3)
O1—Er1—O2	69.20 (9)	O11—N2—O10	121.4 (4)
O5—Er1—O2	143.66 (10)	O11—N2—O9	122.1 (4)
O1—Er1—O10	71.74 (9)	O10—N2—O9	116.5 (3)
O5—Er1—O10	80.73 (10)	C5—O5—Er1	137.4 (2)
O2—Er1—O10	135.43 (9)	C5—O5—H5	107.9
O1—Er1—O7	128.62 (10)	Er1—O5—H5	113.8
O5—Er1—O7	75.93 (10)	O5—C5—C6	110.6 (4)
O2—Er1—O7	67.89 (9)	O5—C5—H5A	109.5
O10—Er1—O7	156.65 (10)	C6—C5—H5A	109.5
O1—Er1—O12	72.33 (10)	O5—C5—H5B	109.5
O5—Er1—O12	73.94 (10)	C6—C5—H5B	109.5
O2—Er1—O12	118.51 (9)	H5A—C5—H5B	108.1
O10—Er1—O12	66.91 (10)	N3—O12—Er1	97.7 (2)
O7—Er1—O12	105.52 (10)	C5—C6—H6A	109.5
O1—Er1—O6	141.92 (10)	C5—C6—H6B	109.5
O5—Er1—O6	74.32 (10)	H6A—C6—H6B	109.5
O2—Er1—O6	80.93 (10)	C5—C6—H6C	109.5
O10—Er1—O6	121.09 (10)	H6A—C6—H6C	109.5
O7—Er1—O6	52.40 (10)	H6B—C6—H6C	109.5
O12—Er1—O6	145.13 (10)	C2—O2—Er1	110.2 (2)
O1—Er1—O3	68.68 (9)	C2—O2—H2	106.7
O5—Er1—O3	132.20 (9)	Er1—O2—H2	113.6
O2—Er1—O3	64.73 (9)	C1—O1—Er1	118.3 (2)
O10—Er1—O3	81.76 (9)	C1—O1—H1	106.9
O7—Er1—O3	114.57 (10)	Er1—O1—H1	120.9
O12—Er1—O3	135.89 (10)	C3—O3—Er1	117.9 (2)
O6—Er1—O3	77.59 (9)	C3—O3—H3	109.0
O1—Er1—O13	74.73 (9)	Er1—O3—H3	117.2
O5—Er1—O13	96.36 (10)	O3—C3—C2	107.0 (3)
O2—Er1—O13	72.82 (9)	O3—C3—C4	111.3 (3)
O10—Er1—O13	116.15 (9)	C2—C3—C4	113.0 (3)
O7—Er1—O13	66.70 (10)	O3—C3—H3A	108.5
O12—Er1—O13	51.66 (9)	C2—C3—H3A	108.5
O6—Er1—O13	118.97 (10)	C4—C3—H3A	108.5
O3—Er1—O13	131.19 (9)	O2—C2—C3	103.6 (3)
O1—Er1—O9	107.95 (9)	O2—C2—C1	107.5 (3)
O5—Er1—O9	70.35 (9)	C3—C2—C1	116.3 (3)
O2—Er1—O9	125.27 (9)	O2—C2—H2A	109.7
O10—Er1—O9	50.86 (9)	C3—C2—H2A	109.7
O7—Er1—O9	119.40 (10)	C1—C2—H2A	109.7
O12—Er1—O9	111.18 (10)	C4—O4—H4	109.4
O6—Er1—O9	70.51 (10)	O1—C1—C2	108.6 (3)
O3—Er1—O9	64.11 (9)	O1—C1—H1A	110.0
O13—Er1—O9	161.77 (10)	C2—C1—H1A	110.0
N2—O9—Er1	92.5 (2)	O1—C1—H1B	110.0
N3—O13—Er1	94.9 (2)	C2—C1—H1B	110.0
N1—O6—Er1	95.4 (2)	H1A—C1—H1B	108.4
N2—O10—Er1	100.1 (2)	O4—C4—C3	111.7 (3)
O8—N1—O7	121.6 (4)	O4—C4—H4A	109.3
O8—N1—O6	122.4 (4)	C3—C4—H4A	109.3
O7—N1—O6	116.0 (3)	O4—C4—H4B	109.3
N1—O7—Er1	96.1 (2)	C3—C4—H4B	109.3
O14—N3—O12	122.8 (4)	H4A—C4—H4B	107.9
O14—N3—O13	122.0 (4)		

### Hydrogen-bond geometry (Å, º)

**Table:**

*D*—H···*A*	*D*—H	H···*A*	*D*···*A*	*D*—H···*A*
O1—H1···O4^i^	0.84	1.83	2.666 (4)	173
O2—H2···O9^ii^	0.84	1.97	2.795 (4)	167
O3—H3···O13^iii^	0.84	2.08	2.917 (4)	175
O4—H4···O11^iv^	0.84	2.07	2.897 (5)	169
O5—H5···O8^v^	0.84	2.09	2.865 (4)	154

Symmetry codes: (i) −*x*+1, −*y*+2, −*z*+1; (ii) −*x*+1, *y*−1/2, −*z*+1/2; (iii) −*x*+1, *y*+1/2, −*z*+1/2; (iv) *x*−1, *y*, *z*; (v) −*x*+1, −*y*+2, −*z*.

## References

[bb1] Bekoe, A. & Powell, H. M. (1959). *Proc. R. Soc. London Ser. A*, **250**, 301–315.

[bb2] Gyurcsik, B. & Nagy, L. (2000). *Coord. Chem. Rev.* **203**, 81–148.

[bb3] Hua, X.-H., Xue, J.-H., Yang, L.-M., Xu, Y.-Z. & Wu, J.-G. (2013). *Acta Cryst.* E**69**, m162–m163. 10.1107/S160053681300305X PMC3588509 23476505

[bb4] Rigaku (2007). *CrystalClear* Rigaku Inc., Tokyo, Japan.

[bb5] Sheldrick, G. M. (2008). *Acta Cryst.* A**64**, 112–122. 10.1107/S0108767307043930 18156677

[bb6] Yang, L.-M., Hua, X.-H., Xue, J.-H., Pan, Q.-H., Yu, L., Li, W.-H., Xu, Y.-Z., Zhao, G.-Z., Liu, L.-M., Liu, K.-X., Chen, J.-E. & Wu, J.-G. (2012). *Inorg. Chem.* **51**, 499–510. 10.1021/ic2019605 22148886

[bb7] Yang, L.-M., Su, Y.-L., Xu, Y.-Z., Wang, Z.-M., Guo, Z.-H., Weng, S.-F., Yan, C.-H., Zhang, S.-W. & Wu, J.-G. (2003). *Inorg. Chem.* **42**, 5844–5856. 10.1021/ic0300464 12971752

[bb8] Yang, L.-M., Su, Y.-L., Xu, Y.-Z., Zhang, S.-W., Wu, J.-G. & Zhao, K. (2004). *J. Inorg. Biochem.* **98**, 1251–1260. 10.1016/j.jinorgbio.2004.05.012 15271500

